# Fluid shear stress induces cell migration and invasion via activating autophagy in HepG2 cells

**DOI:** 10.1080/19336918.2019.1568141

**Published:** 2019-01-31

**Authors:** Zhiping Yan, Guanyue Su, Wenbo Gao, Jia He, Yang Shen, Ye Zeng, Xiaoheng Liu

**Affiliations:** Institute of Biomedical Engineering, West China School of Basic Medical Sciences & Forensic Medicine, Sichuan University, Chengdu, China

**Keywords:** Fluid shear stress, autophagy, integrin, cytoskeleton, metastasis

## Abstract

Fluid shear stress (FSS) regulates the metastasis of hepatocellular carcinoma (HCC). In the present study, we aimed to study the role of autophagy in HCC cells under FSS. The results showed that FSS upregulated the protein markers of autophagy, induced LC3B aggregation and formation of autophagosomes. Inhibition of integrin by Cliengitide (Cli) or inhibition of the microfilaments formation both inhibited the activation of autophagy in HepG2 under FSS. In addition, Cli inhibited the microfilaments formation and expressions of Rac1 and RhoA in HepG2 cells under FSS. Finally, inhibition of autophagy suppressed the cell migration and invasion in HepG2 under FSS. In conclusion, FSS induced autophagy to promote migration and invasion of HepG2 cells via integrin/cytoskeleton pathways.

## Introduction

Hepatocellular carcinoma (HCC) accounts >90% cases of primary liver cancers, which occurred predominantly in the patients with chronic hepatitis B and cirrhosis [1]. The mortality of advanced HCC is still high [2]. One reason is the high metastasis capacity of the advanced HCC. Studying on the molecular mechanism underlying the metastasis of HCC is an important way to develop new strategy in tumor therapy [3].

HCC is generally hypervascular, and flow in the vascular vessels promotes the development and metastasis of HCC [4]. The fluid shear stress (FSS) due to flow in normal physiological liver tissues is less than 0.1 dyn/cm^2^ [5]. In the HCC, the biophysical microenvironment is changed with the tissue reorganization [6]. The mechanical stiffness and pressure in the HCC native tissues were obviously higher than the healthy liver tissues [7]. The interstitial fluid pressure gradient in the healthy liver tissues between tumor and stroma was found to be 2.2 mmHg, while it in a hepatoma was ranged 0–30 mmHg [8]. A pressure gradient drives interstitial fluid flow through the tumor stroma. It was demonstrated that the velocity of interstitial fluid flow was ranged from 0.1 to 6.0 μm/s in the published in vivo and in vitro study [9]. Qazi et al. found that FSS at 0.84 and 2.53 dyn/cm^2^ enhanced the metastatic potential of metastatic cell [10,11]. We previously demonstrated that FSS induced the changes in cell morphology and migration capacity of cancer cells including laryngeal squamous cell carcinoma Hep-2 cells and HCC HepG2 cells [12,13]. FSS at 1.4 dyn/cm^2^ induced the epithelial-mesenchymal transition of Hep-2 cells through integrin-ILK/PI3K-AKT-Snail signaling pathway [12], which might contribute to cancer metastasis [14,15]. In HepG2 cells, FSS at 1.4 dyn/cm^2^ induced cell migration through Integrins- FAK-Rho GTPases (Cdc42, Rac1, and RhoA) signaling pathway [13].

In recent, it was demonstrated that autophagy plays an important role in cancer metastasis [16]. FSS could induce autophagy in various cancer cells, such as cervical cancer cells and HCC cells [17,18]. Das et al. [17] found that the physiological level of FSS (2 Pa) induced autophagy in the cervical cancer cell line HeLa, which inhibited the early onset of apoptosis. Lien et al. [18] reported that FSS (0.5, 6, and12 dyn/cm^2^) induced acidic vesicular organelle formation, microtubule-associated protein light chain 3 (LC3B) transformation and p62/SQSTM1 degradation in the HCC Hep3B cells, suggesting FSS induced autophagy in the HCC cells. However, the molecular mechanism by which FSS-induced autophagy in the HCC cells was remained unclear.

It was proposed that cytoskeleton plays an essential role in mediating the autophagy flux [19]. Aguilera et al. [20] had demonstrated that actin cytoskeleton is necessary for the starvation induced autophagy. The depolymerization of actin filament by latrunculin B (LaB) inhibited the autophagic vacuoles formation induced by starvation stimuli [20]. It was observed colocalization between actin fibers and Atg14, Beclin-1 in reticular-like structures in the cytoplasm and in some punctate structures close to the plasma membrane, indicating that actin is involved in very early stage of autophagosome formation [20].

Rho GTPases regulated actin-based processes [21]. The members of Rho family including Cdc42, Rac1 and RhoA are well characterized and have different regulatory functions in the cytoskeleton remodeling. RhoA controls the formation of stress fibers and contractile ring, whereas Cdc42 and Rac1 modulate the formation of lamellipodia and filopodia [22]. In starvation administrated HeLa cells, RhoA activates but Rac1 inhibits the autophagy [20]. Both RhoA and Rac1 triggers a series of cellular signals that mutually regulated the autophagy [20]. In addition, Rho GTPases (Cdc42, Rac1 and RhoA) interact with integrins in the mechanotransduction of HepG2 [13]. It was reported that autophagy is closely associated with the cancer stem-like phenotype promotion via integrin αvβ3 pathway in HCC cell [23], indicating a regulatory role of integrin in autophagy. However, the role of integrin-cytoskeleton signaling pathway in FSS-induced autophagy, and its effect on migration and invasion of HCC cells are still unknown.

In the present study, we observed FSS-induced formation of autophagosome in HCC cells in the presence of FSS, analyzed the roles of integrin and cytoskeleton in the activation of autophagy, and detected the role of autophagy in the FSS-induced migration and invasion of HCC cells. The results showed that FSS induced autophagy in HepG2 cells through the integrin-cytoskeleton signaling pathway, and that inhibition of autophagy suppressed the migration and invasion of HCC cells, which might provide a novel treatment strategy for HCC.

## Results

### FSS induced autophagy in HepG2 cells

**Figure 1.**
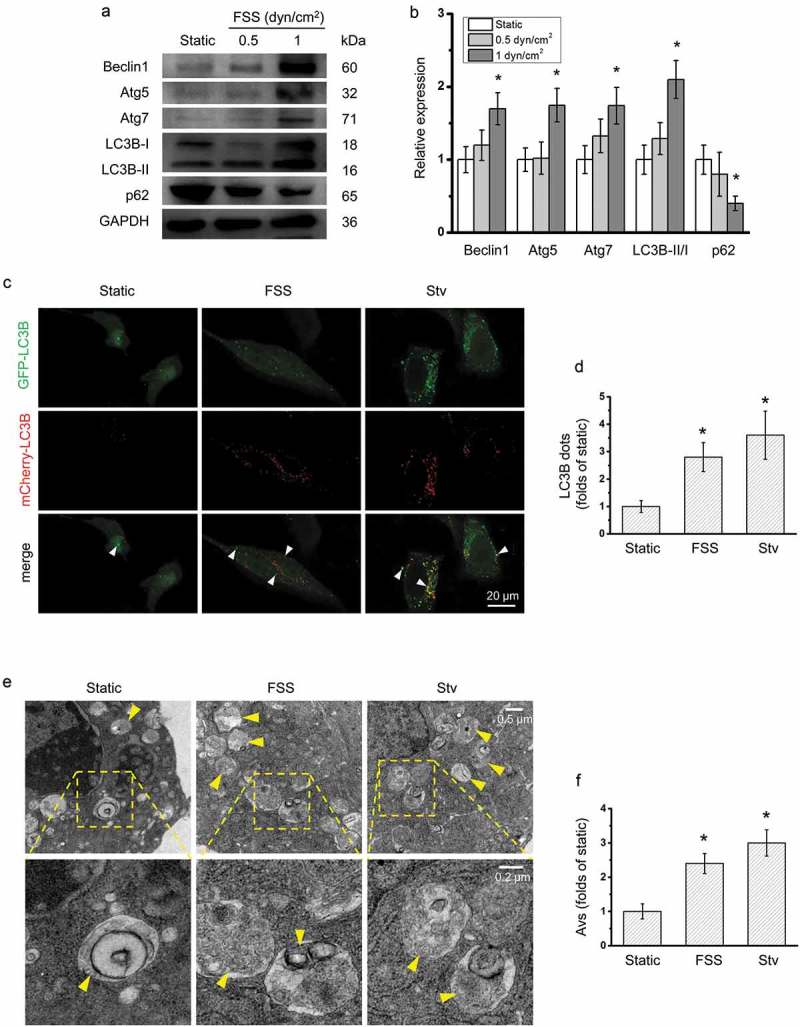
FSS activated autophagy in HepG2 cells. (a) HepG2 cells were exposed to FSS at 0.5 or 1 dyn/cm^2^ for 0.5 h, or treated with Earle’s balanced salt solution (EBSS) for 2 h. Lysates were probed with antibodies as indicated. (b) Quantification of protein expressions in (a). (c) After Ad-mCherry-GFP-LC3B transfection, HepG2 cells were loaded with FSS at 1 dyn/cm^2^ for 0.5 h or treated with EBSS for 2 h. Immunostaining of LC3B (Green, GFP-LC3B; Red, mCherry-LC3B) were performed. The white arrow indicated the LC3B punctate dots (yellow dots, autophagosomes). (d) LC3B dots were counted from at least 20 random cells (n = 3). (e) HepG2 cells were loaded with FSS at 1 dyn/cm^2^ for 0.5 h or treated with EBSS for 2 h. Representative images were photographed by TEM. The yellow arrow indicated the autophagic vacuole (AV). (f) AVs were counted from at least 10 random field (25 μm^2^) imaged by TEM (n = 3). *p < 0.05 vs. static.

During autophagy, a series of autophagic markers such as Beclin-1, Atg5, Atg7 and LC3B-II associated with the formation of autophagosome are largely activated [16]. LC3B-II is localized and aggregated in autophagosomes. The transformation of LC3B-I to LC3B-II (the lipidated form of LC3B) has been considered to be a characteristic of autophagy. In addition, p62 and p62-bound polyubiquitinated proteins become incorporated into the completed autophagosome and are degraded in autolysosomes, thus p62 was served as an index of autophagic degradation [24]. To examine the autophagy in HepG2 cells applied with FSS, we detected the expression of Beclin-1, Atg5, Atg7, LC3B and p62 using western blotting (Figure 1(a)). FSS at 1 dyn/cm^2^ for 0.5 h significantly upregulated the expression of Beclin-1, Atg5, Atg7, ratio of LC3B-II/I and downregulated the expression of p62 in HepG2 cells (Figure 1(a,b)).

For the detection of the autophagosomes formation, HepG2 cells were transfected with the Ad-mCherry-GFP-LC3B and the LC3B punctate dots and autophagic vacuoles were observed by confocal microscopy and TEM. Like in the serum starvation condition (positive control), LC3B punctate dots in the cytoplasm were significantly increased by 1 dyn/cm^2^ FSS for 0.5 h (Figure 1(c,d)). Also, FSS significantly induced formation of autophagic vacuoles, compared with static control (Figure 1(f)). All these results suggested that FSS at 1 dyn/cm^2^ for 0.5 h induced autophagy in HepG2.

### Integrin involved in the FSS-induced autophagy in HepG2 cells

**Figure 2.**
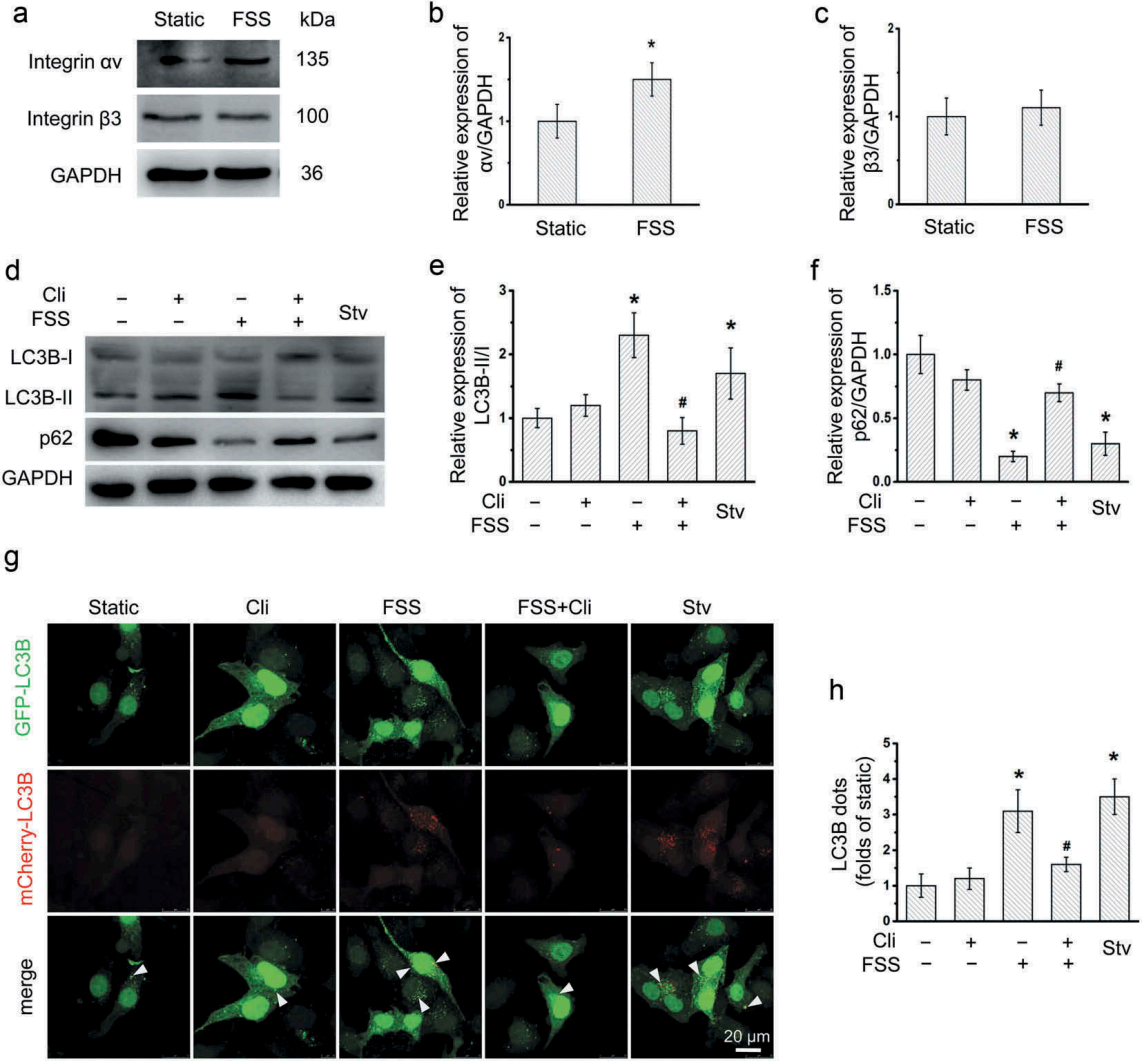
Inhibition of integrin attenuated FSS-activated autophagy in HepG2 cells. (a) HepG2 cells were loaded with FSS at 1 dyn/cm^2^ for 0.5 h. Lysates were probed with antibodies as indicated. (b and c) Quantification of integrin αv and Integrin β3 in (a). (d) HepG2 cells were loaded with FSS at 1 dyn/cm^2^ for 0.5 h with or without treatment of 0.5 µM Cliengitide (Cli) for 6 h prior to FSS application. Lysates were probed with antibodies as indicated. (e and f) Quantification of protein expression in (d). (g) After Ad-mCherry-GFP-LC3B transfection, HepG2 cells were loaded with FSS at 1 dyn/cm^2^ for 0.5 h, with or without treatment of 0.5 µM Cliengitide (Cli) for 6 h prior to FSS application. Immunostaining of LC3B (Green, GFP-LC3B; Red, mCherry-LC3B) were performed. The white arrow indicated the LC3B punctate dots (yellow dots, autophagosomes). (h) LC3B dots were counted from at least 20 random cells (n = 3). *p < 0.05 vs. Static; ^#^*p* < 0.05 vs. FSS.

Previous studies have shown that integrin plays an important role in the mechanotransduction of FSS [25]. The expression of integrin subunits αv and β3 in HepG2 cells applied to FSS were detected by western blotting (Figure 2(a–c)). FSS significantly upregulated the expression of Integrin αV, but not β3. To confirm the role of integrin in FSS-induced autophagy, HepG2 cells were treated with integrin αVβ3 inhibitor Cli in the presence of FSS. The ratio of LC3B-II/I was significantly inhibited by Cli in HepG2 cells in the presence of FSS, compared with FSS alone (Figure 2(d,e)). The expression of p62 was significantly downregulated by FSS (Figure 2(d,f)). However, in the presence of Cli, the p62 expression was not significantly changed by FSS (Figure 2(d,f)).Moreover, the LC3B punctate dots were significantly reduced by Cli in Ad-mCherry-GFP-LC3B-transfected HepG2 cells under FSS compared with FSS alone (Figure 2(g,h)). These results suggested that FSS-induced autophagy in HepG2 via integrin pathway.

### Actin cytoskeleton involved in the FSS-induced autophagy in HepG2 cells

**Figure 3.**
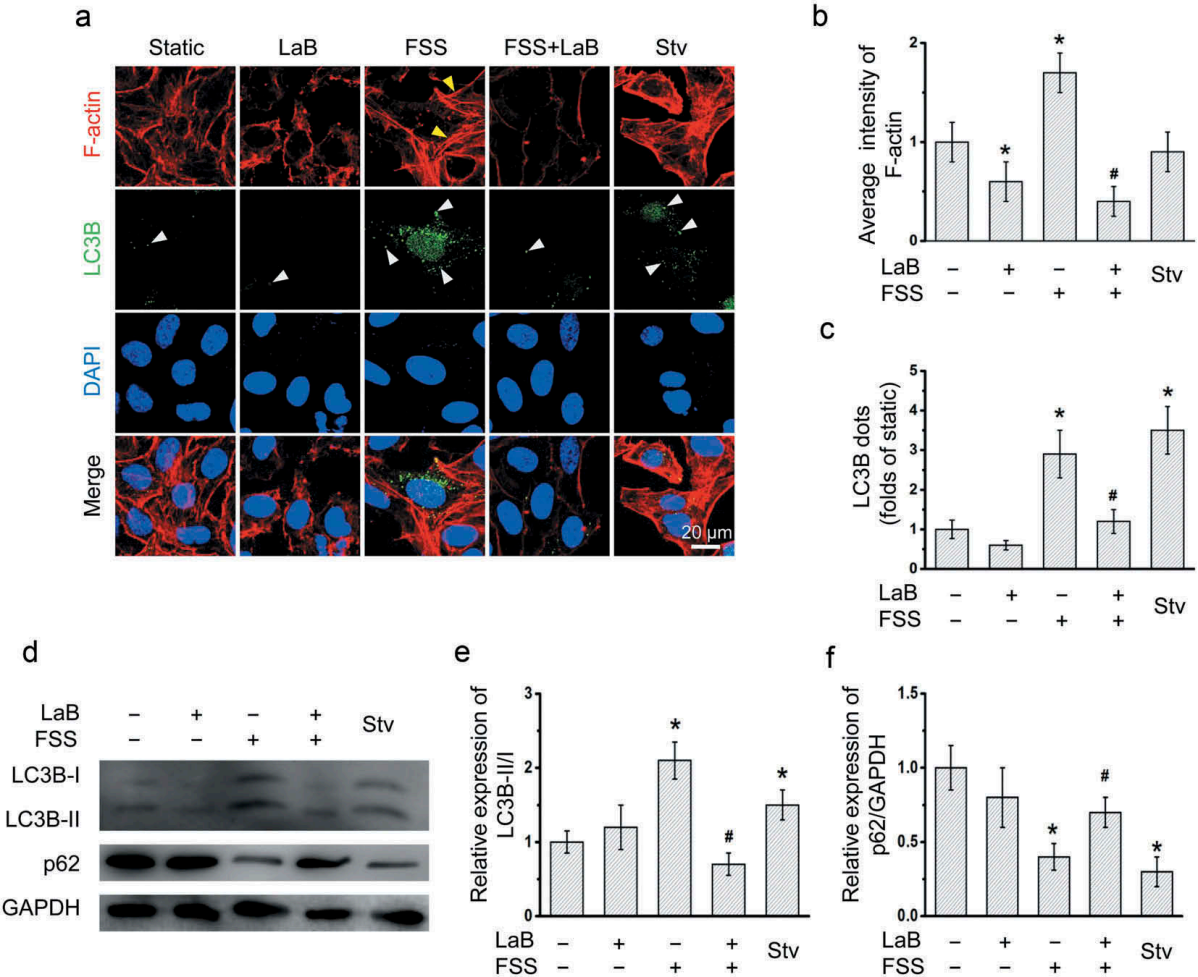
Inhibition of actin microfilament polymerization attenuated FSS-induced autophagy in HepG2 cells.(a) After Ad-mCherry-GFP-LC3B transfection, HepG2 cells were loaded with FSS at 1 dyn/cm^2^ for 0.5 h, with or without treatment of 10 μM Latrunculin B (LaB) for 2 h prior to FSS application. Immunostaining of F-actin (Red), nuclei (Blue), and LC3B (Green) were performed. The white arrow indicated the LC3B punctate dots. (b) The average intensity of F-actin was analyzed from at least 10 random field (4*10^4^ μm^2^) (n = 3). (c) LC3B dots were counted from at least 20 random cells (n = 3). (d) HepG2 cells were loaded with FSS at 1 dyn/cm^2^ for 0.5 h, with or without treatment of 10 μM LaB for 2 h prior to FSS application. Lysates were probed with antibodies as indicated. (e and f) Quantification of LC3B-II/I and p62 in (d). *p < 0.05 vs. Static; ^#^*p* < 0.05 vs. FSS.

To explore the role of actin cytoskeleton in the FSS-induced autophagy, we pretreated the HepG2 cells with a microfilament polymerization inhibitor LaB (Figure 3). Using immunofluorescence and confocal microscopy, it was observed that the average intensity of actin microfilaments and LC3B punctate dots were significantly reduced by LaB in FSS-applied HepG2 cells (Figure 3(a–c)). In the presence of FSS, the ratio of LC3B-II/I was also significantly inhibited by LaB, while the p62 expressions was significantly increased by LaB (Figure 3(d–f)). These results suggested that actin microfilaments play a vital role in the FSS-induced autophagy in HepG2 cells.

### Integrin was associated with the actin cytoskeleton in HepG2 cells

**Figure 4.**
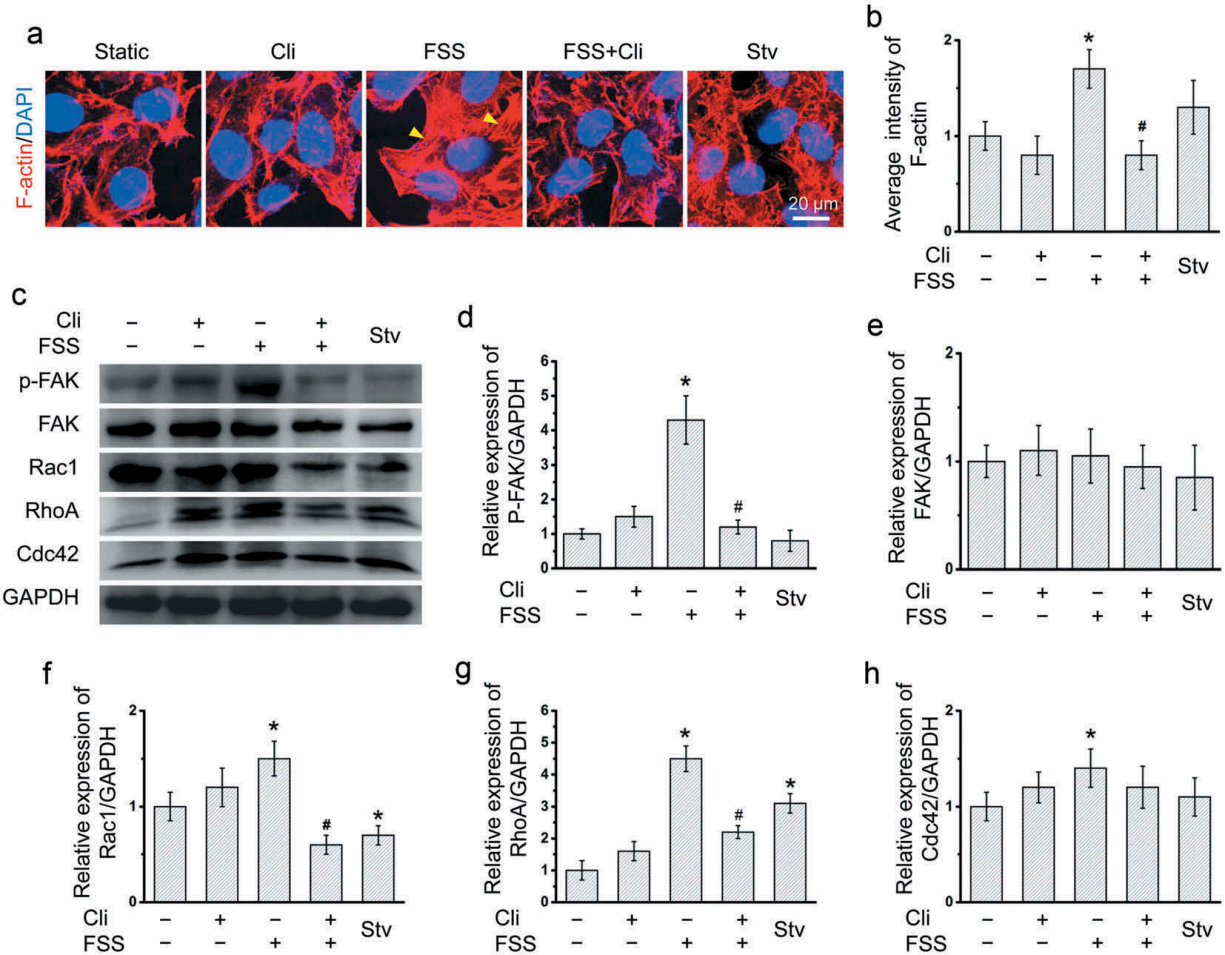
Inhibition of integrin attenuated FSS-induced cytoskeleton rearrangement. (a) HepG2 cells were loaded with FSS at 1 dyn/cm^2^ for 0.5 h, with or without treatment of 0.5 µM Cliengitide (Cli) for 6 h prior to FSS application. Immunostaining of F-actin (Red), nuclei (Blue) were performed. The yellow arrow indicated the stress fibers. (b) The average intensity of F-actin was analyzed from at least 10 random field (4*10^4^ μm^2^) (n = 3). (c) HepG2 cells were loaded with FSS at 1 dyn/cm^2^ for 0.5 h, with or without treatment of 0.5 µM Cli for 6 h prior to FSS application. Lysates were probed with antibodies as indicated. (d-g) Quantification of protein expressions in (c). *p < 0.05 vs. Static; ^#^*p* < 0.05 vs. FSS.

The activation of FAK was involved in integrin-mediated cell signaling in various epithelial cancers [26]. In HepG2 cells, FSS also significantly induced cytoskeleton rearrangement (Figure 4(a,b)) and FAK activation (Figure 4(c–e)). In the presence of FSS, inactivation of integrin by Cli significantly inhibited the cytoskeleton rearrangement and FAK activation in HepG2 cells.

Rho GTPases including RhoA, Rac1 and Cdc42 play important roles in cytoskeleton rearrangement, cell motility and adhesion [21,22]. The expressions of RhoA, Rac1 and Cdc42 were significantly enhanced by FSS (Figure 4(c,f-h)). Inactivation of integrins by Cli significantly inhibited the increase of RhoA and Rac1 expressions by FSS (Figure 4(c,f,g)). These results suggested that FSS induced cytoskeleton rearrangement in HepG2 cells via integrin signaling pathway.

### Inhibition of autophagy by 3-MA and lentivirus-derived Atg5 shRNA in HepG2 cells under FSS

**Figure 5.**
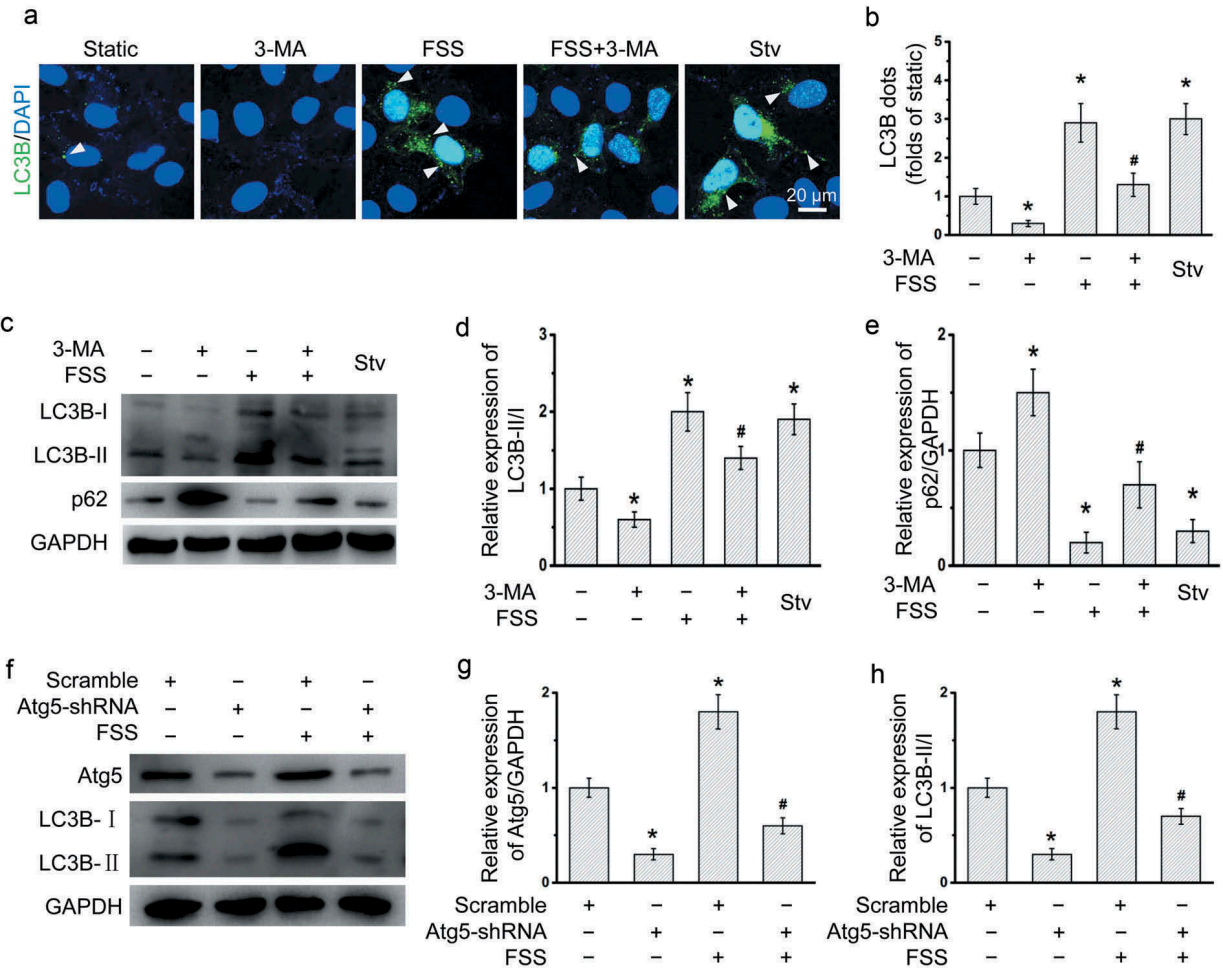
Inhibition autophagy by 3-MA or lentivirus-delivered Atg5 shRNA in HepG2 cells under FSS. (a) After Ad-mCherry-GFP-LC3B transfection, HepG2 cells were loaded with FSS at 1 dyn/cm^2^ with or without treatment of 5 mM 3-MA for 12 h. Immunostaining of nuclei (Blue), and LC3B (Green) were performed. The yellow arrow indicated the LC3B punctate dots. (b) LC3B dots were counted from at least 20 random cells (n = 3). (c) HepG2 cells were loaded with FSS at 1 dyn/cm^2^ with or without treatment of 5 mM 3-MA for 12 h. (d and e) Quantification of LC3B-II/I and p62 in (c). (f) HepG2 cells were loaded with FSS at 1 dyn/cm^2^ with transfection of lentivirus-delivered scramble or Atg5 shRNAs for 12 h. (g-i) Quantification of Atg5 and LC3B in (f). *p < 0.05 vs. Static; ^#^*p* < 0.05 vs. FSS.

3-MA was widely used to inhibit the autophagy. Atg5 functions as an E1-like activating enzyme in a ubiquitin-like conjugating system, involving in the autophagic vesicle formation [27]. To inhibit the autophagy under FSS, cells were treated with 3-MA or transfected with lentivirus-delivered Atg5 shRNA. With 3-MA treatment in the presence of FSS, the LC3B punctate dots was significantly reduced in HepG2 cells compared with FSS (Figure 5(a,b)), the ratio of LC3B-II/I was significantly inhibited (Figure 5(c–e)), and the p62 expression was significantly upregulated (Figure 5(c–e)). Atg5 shRNA significantly downregulated the expression of Atg5 in HepG2 cells (Figure 5(f,g)). The ratio of LC3B-II/I was significantly inhibited by Atg5 shRNA in the presence of FSS. Thus, autophagy in HepG2 cells under FSS could be inhibited by 3-MA and Atg5 shRNA.

### FSS induced the migration and invasion of HepG2 cells via the autophagy induction

**Figure 6.**
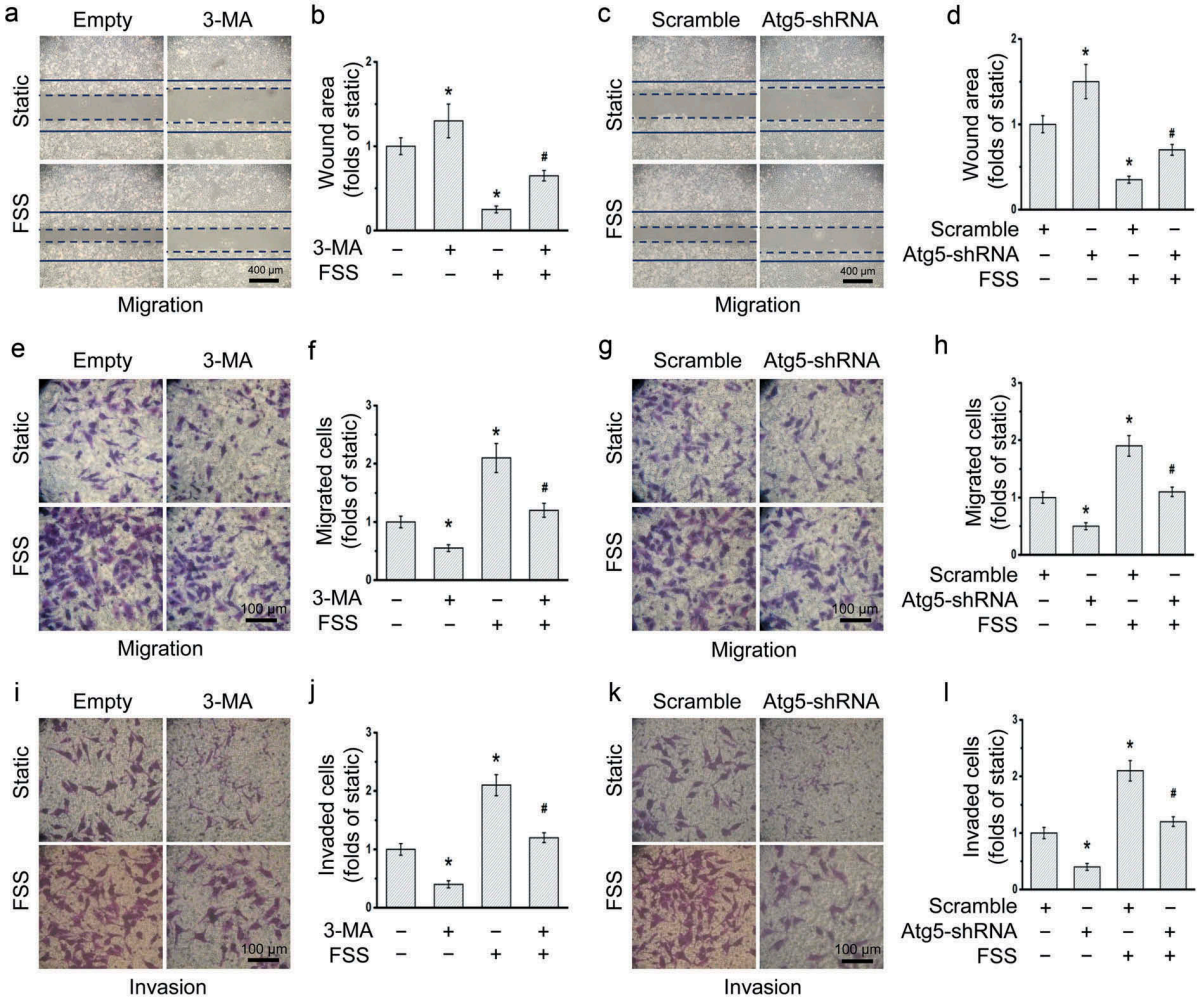
Autophagy inhibition attenuated FSS-induced migration and invasion in HepG2 cells. HepG2 cells were loaded with FSS at 1 dyn/cm^2^ with or without treatment of 5 mM 3-MA for 12 h (a-b, e-f, and i-j), or with or without transfection of lentivirus-delivered scramble or Atg5 shRNAs for 12 h (c-d, g-h, and k-l). (a, c) Representative images of wound area were photographed. The solid line represents the initial wound, and the dashed line represents the final wound after treatment. The FSS direction was perpendicular to the solid lines (the scratch wound). (B, D) Quantification of wound areas in (a,c) at least 10 random field (1.6*10^4^ μm^2^) (n = 3). (e, g) Representative images of cell migration of FSS-applied cells were photographed by Transwell migration assay. (f, h) Quantification of migrated cells in (e,g). (i, k) Representative images of cell invasion of FSS-applied cells were photographed by Transwell invasion assay. (j, l) Quantification of invaded cells in (i,k). *p < 0.05 vs. Static; ^#^*p* < 0.05 vs. FSS.

The role of autophagy in the FSS-induced migration and invasion of HepG2 cells were detected (Figure 6). After treated with 3-MA or lentivirus-derived Atg5 shRNA, the FSS-induced migration of HepG2 cells were significantly reduced with an increased wound area (Figure 6(a–d)).

The migration and invasion capacities of the cells after FSS application for 24 h were also detected by Transwell assays (Figure 6(e–l)). FSS-applied cells showed increase in migration and invasion capacities. In the presence of FSS, both migrated and invaded cells were significantly reduced after inhibition of autophagy by 3-MA or Atg5 shRNA. These results suggested that FSS induced the migration and invasion of HepG2 cells via the autophagy activation.

Overall, FSS induced migration and invasion of HepG2 cells through the integrin-cytoskeleton mediated autophagy activation.

## Discussion

The incidence of HCC has increased sharply, second only to lung and gastric cancers in China [2]. FSS in the tumor microenvironment plays crucial roles in the growth, invasion, and metastasis of cancer cells [11]. Here, we explored the mechanism in the FSS-induced HCC migration.

Recent evidence suggest that autophagy has a protective function in limiting genome damage, necrosis and inflammation in tumor in response to metabolic stress [28]. Autophagy is characterized by formation of autophagic vesicles and maturation of autophagosome [24]. The classic Akt-mTOR signaling pathway plays a typical negative regulatory role in the initiation formation of the vesicular double-membrane. The Beclin-1/class III PI3-Kinase (PI3K) complexes including Beclin-1, PI3K Vacuolar protein sorting 34 (Vps34, target of 3-MA) could be recruited to the isolated membrane to initialize the nucleation and elongation of autophagosome. The mature autophagic vesicles were fused with lysosomes. Their maturation is depended on two separate autophagy-related gene (Atg) ubiquitin-like conjugation systems including the Atg5-12 conjugation system and Atg8/LC3 conjugation system [29]. Atg5 functions as an E1-like activating enzyme in the ubiquitin-like conjugating system, which direct participated in the expansion of autophagic membrane [27]. LC3B is expressed as a full-long cytosolic protein in most cell types, and is proteolytically cleaved by a cysteine protease Atg4 to generate LC3B-I upon induction of autophagy. LC3B-II is found on both the internal and external surfaces of autophagosome, which plays an important role in membranes hemifusion and selecting cargo for degradation [30]. In the present study, application of 1 dyn/cm^2^ FSS for 0.5 h significantly upregulated the expressions of autophagic markers including Beclin-1, Atg5, Atg7, and ratio of LC3B-II/I in HepG2 cells. Also, FSS induced LC3B aggregation and formation of autophagosomes (Figure 1). Thus, FSS induced autophagy in HepG2 cells.

Our previous works showed that FSS regulated cell function via changing the morphology and inducing rearrangement of cytoskeleton in a variety of cells, such as endothelial cell, HCC cells [12,31]. It was demonstrated that actin cytoskeleton plays an important role in starvation-induced autophagy [20]. Actin filaments and autophagic markers such as Atg14 and Beclin-1 was colocalized in starved Hela cell, while LaB induced depolymerization of actin filaments and then inhibited the formation of autophagic vacuoles in response to the starvation stimulus [20]. Our results confirmed that actin cytoskeleton plays an important role in autophagy activation in the presence of FSS. LaB inhibited the formation of autophagic vesicles, and reduced the expression of autophagy markers and ratio of LC3B-II/I in HepG2 cells (Figure 3).

**Figure 7.**
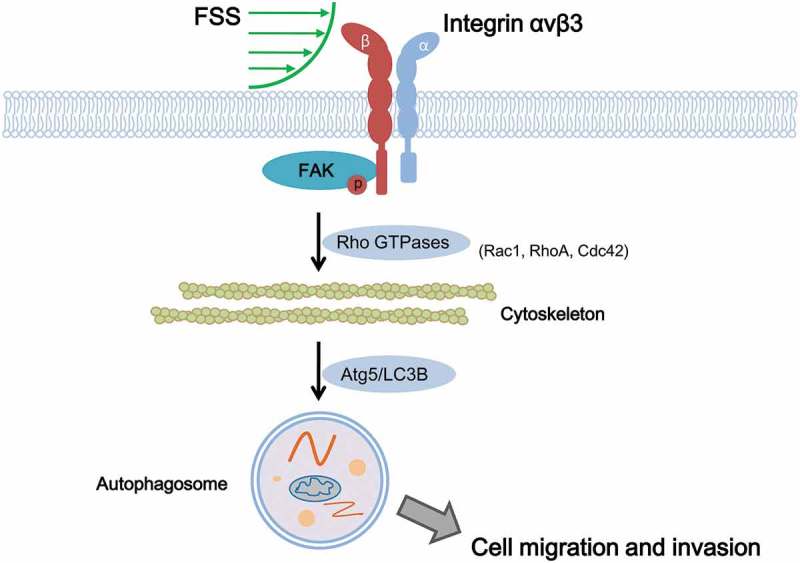
Schematic illustrating mechanical mechanism of FSS induced cell migration and invasion in HepG2 cells. FSS induced the expressions of RhoGTPases including Rac1, RhoA and Cdc42 and the cytoskeleton remodeling via integrin/FAK pathway, leading to upregulation of Atg5 and LC3B II/I to activate autophagy. Autophagy plays important role in FSS-induced cell migration and invasion in HepG2 cells.

Integrin serves as an important mechanosenor for FSS [25]. We observed that integrin inhibitor Cli inhibited activation of downstream FAK and attenuated FSS-induced activation of autophagy in HepG2 cells (Figure 2). In addition, Cli inhibited polymerization of actin microfilaments under FSS (Figure 4). The rearrangement of cytoskeleton is dependent on RhoGTPases [21]. FSS induced expressions of RhoGTPases including RhoA, Cdc42, and Rac1 in HepG2 cells [13]. We further showed that inactivation of integrin by Cli inhibited the expression of RhoA and Rac1 (Figure 4), which further strengthened the association between integrin signaling and actin cytoskeleton in FSS-induced autophagy. Integrin αV could bind to β3 subunit [32]. The molecular conformation of β3 subunit undergoes change under FSS, thus to activate FAK [33]. Our results showed that FSS induced expression of integrin αV and FAK activation, suggesting FSS induced the autophagy via integrin-cytoskeleton signaling pathway (Figure 7).

FSS induced epithelial-mesenchymal transition in Hep-2 cells [12]. FSS induced migration of HepG2 cells [13]. FSS also induced invasion of HepG2 cells. Inhibition of autophagy by 3-MA and lentivirus-derived Atg5 shRNA (Figure 5) suppressed the cell migration and invasion in presence of FSS (Figure 6), suggesting FSS plays an important role in metastasis of cancer cells through autophagy pathway. Autophagy is important for maintaining cellular homeostasis by removing protein aggregates and destructing cellular organelles, as well as recycling the by-products of autophagic degradation [16]. Increasing evidences demonstrated that autophagy directly involved in tumor cell motility and invasion during metastasis, in part through turnover of components of the cell migration machinery such as focal adhesions [34]. Our study suggested that FSS induced activation of FAK, which might associate with the turnover of focal adhesions.

The study has some limitations. For example, the effects of RhoGTPases on cytoskeleton remodeling and autophagy activation should be confirmed by RNA interference tools. The effect of FSS in metastasis of HCC cells in vivo is difficult to evaluate. These limitations should be investigated in the future.

In summary, FSS induced the expressions of RhoGTPases and the cytoskeleton remodeling via integrin/FAK pathway, leading to upregulation of Atg5 and LC3B II/I to activate autophagy. Autophagy plays important role in FSS-induced cell migration and invasion in HepG2 cells (Figure 7). Thus, FSS induced migration and invasion of HepG2 cells through the integrin-cytoskeleton-mediated autophagy activation. We presently validated the induction of autophagy by FSS, and the mechanical mechanism regarding integrin and microfilament in mediation of autophagy in HCCs, which is helpful to understand the mechanism of mechanical microenvironment in regulating cancer metastasis and to develop novel drugs for HCC treatment.

## Materials and methods

### Cell culture

HepG2 cells (American Type Culture Collection, USA) were cultured in RPMI1640 (22400089, Invitrogen, USA) supplemented with 10% fetal bovine serum (FBS), 100 U/L penicillin and 100 mg/L streptomycin (SV30010, Hycone, USA) at 37°C in an atmosphere containing 5% CO_2_.

### Gene silencing of Atg5 with lentivirus-delivered Atg5 shRNA

HepG2 cells were transfected with the recombinant virus (LV-ATG5-RNAi, 9514–1, GeneChem, China) to inhibit the autophagy. The oligonucleotides encoding the Atg5-shRNA or scramble-shRNA sequence were inserted into the EGFP express vector GV248 (GeneChem, China). The Atg5 shRNA sequence is 5ʹ-CCTTTCATTCAGAAGCTGTTT-3ʹ. The scrambled shRNA sequence is 5ʹ-TTCTCCGAACGTGTCACGT-3ʹ, which was used as a negative control. After transfection for 3 days, the EGFP-positive cells were counted under fluorescence microscope to determine transfection efficiency. In addition, the Atg5 expression were determined by western blotting analysis.

### FSS application

HepG2 cells were dissociated enzymatically with 0.25% trypsin, and then resuspended in culture medium containing 10% fetal bovine serum. Cell density at 1.0 × 10^5^ cells/mL was determined with a haemocytometer and seeded on the sterilized glass slide (24 mm × 75 mm) in the polystyrene tissue culture plate. Until cell reached 95% confluence on the glass slides, the HepG2 cells were exposed to 0.5, 1 dyn/cm^2^ FSS using a parallel flow chamber [35,36]. The static cultured HepG2 cells without FSS stimulation were set as the control group. For inhibitor application, HepG2 cells were pretreated with 0.5 µM integrin inhibitor Cliengitide (Cli, HY-16141, MedChemExpress, USA) for 6 h, 10 µM microfilament polymerization inhibitor Latrunculin B (LaB, ab144291, abcam, UK) for 2 h, or 5 mM autophagy inhibitor 3-Methyladenine (3-MA, M9281, Sigma, USA) for 12 h, and was also added in perfusion medium.

### Western blotting analysis

Cells were washed and then lysed on ice for 30 min using RIPA Lysis Buffer (P0013C, Beyotime, China) with an addition of protease inhibitor cocktail (BB3301-1, BestBio Science, China), phosphatase inhibitor cocktail (BB3311-1, BestBio Science, China) and phenylmethylsulfonyl fluoride (ST506-1, Beyotime, China) in 1:100. Protein concentration was measured by a Protein Determination Kit (704002, Cayman, USA). Protein samples (20 μg) were size fractionated using SDS-PAGE (8–12%) and electrotransferred onto polyvinylidene fluoride membrane (ISEQ00010, Millipore, USA). Membranes were blocked for 2 h with 5% bovine serum albumin (BSA) in Tris-buffered saline with Tween 20 (TBST, 20 mM Tris-HCl, 150 mM NaCl, 0.1% Tween 20) at room temperature. Blots were incubated with the respective primary antibodies overnight at 4°C. GAPDH was used as an internal control. The primary antibodies: rabbit-anti-Beclin 1 (1:200, sc-11427, Santa Cruz, USA), rabbit-anti-Atg5 (1:200, sc-133158, Santa Cruz, USA), rabbit-anti-Atg7 (1:200, sc-33211, Santa Cruz, USA), mouse-anti-SQSTM1/p62 (1:200, sc-28359, Santa Cruz, USA), mouse-anti-GAPDH (1:1000, TA-08, ZSJB-BIO, China), rabbit-anti-LC3B (1:1000, ab58610, SIGMA, USA), rabbit-anti-integrin αV (1:1000, sc-376156, Santa Cruz, USA), and rabbit-anti-integrin β3 (1:1000, #13166, Cell Signaling, USA), mouse-anti-p-FAK (1:200, sc-81493, Santa Cruz, USA), rabbit-anti-FAK (1:200, sc-557, Santa Cruz, USA), mouse-anti-RhoA (1:200, sc-418, Santa Cruz, USA), rabbit-anti-Rac1 (1:200, sc-95, Santa Cruz, USA), mouse-anti-Cdc42 (1:200, sc-8401, Santa Cruz, USA). After washing three times in TBST, blots were incubated with HRP-conjugated anti-rabbit (1:3000, ZB-2301, ZSJB-BIO, China) or anti-mouse (1:3000, ZB-2305, ZSJB-BIO, China) secondary antibodies for 1 h at room temperature. Detection was carried out using enhanced chemiluminescence reagents BeyoECL Plus (P0018, Beyotime, China). Blots were imaged by ChemiDoc^TM^ XRS+ system with Image Lab^TM^ Software (version 3.0, Bio-Rad, USA) and analyzed by ImageJ 1.50b (National Institutes of Health, Washington, D.C., USA) with Gel Analyzer plugin.

### Visualization of LC3B punctate dots and actin microfilaments

HepG2 cells were transfected with a adenovirus expressing mCherry-GFP-LC3B fusion protein (Ad-mCherry-GFP-LC3B) (C3011, Beyotime Bio, China) according to the manufacturer’s instructions. After adenovirus transfection at a multiplicity of infection (MOI) of 20 for 24 h, the adenovirus-containing culture medium was removed and then the fresh complete culture medium was added to each well for another 24 h culture. The transfected cells were visualized using confocal microscope (FV1000, Olympus, Japan). LC3B punctate dots was calculated by using ImageJ 1.50b. For actin microfilaments in HepG2 cells with or without Ad-mCherry-GFP-LC3B transfection, cells were washed in PBS 2 times, fixed with 4% paraformaldehyde for 10 min at room temperature, permeabilized with 0.5% Triton X-100 for 3 min, washed 3 times in PBS, blocked with 1% BSA for 30 min, and stained with Texas Red®-X phalloidin (T7471, Invitrogen, USA) for 30 min and DAPI (4ʹ6′-diamidino-2-phenylindole, 760–4196, Roche, Germany) for 15 min at room temperature. Cells were sealed by 10% glycerol, kept in a dark place. The actin cytoskeleton (red) and nuclei (DAPI, blue) were visualized using confocal microscope (FV1000, Olympus, Japan). The average intensity of F-actin was measured by using ImageJ 1.50b.

### Transmission electron microscopy (TEM)

HepG2 Cells were fixed in 2.5% glutaraldehyde in 0.1 M sodium cacodylate for 2 h, postfixed with 1% OsO4 for 2 h and washed in 0.1 M sodium cacodylate 3 times, 15 min each. Then, samples were dehydrated with graded alcohol (50%, 70%, 90%, 90% ethanol+90% acetone, 90% acetone, 100% acetone; 15 min each). After embedded the samples in low viscosity resin, ultrathin sections were cut by Ultramicrotome (EMUC6, Leica, Germany), and then counterstained with 3% uranyl acetate and lead citrate. The formation of autophagosome was examined by a transmission electron microscope (JEM1230, JEOL, Japan).

### Migration and invasion assays

Both wound healing and Transwell assays were performed. For wound healing assay, HepG2 cells were seeded at a density of 10^5^ cells/mL on the slides until 95% confluence. Wound were created by scraping cells off the slides with a plastic scraper. After exposure to 1 dyn/cm^2^ FSS with or without autophagy inhibition treatment (3-MA or lentivirus-derived Atg5 shRNA) for 12 h, cells were imaged by an invert contrast microscopy (CKX41, Olympus, Japan) and digitized using a digital camera (G11, Cannon, Japan). The wound healing areas were calculated to evaluate the cell migration capacity by using ImageJ 1.50b. For Transwell assays, after autophagy inhibition and FSS application for 12 h, cells were collected and seeded into the upper chamber of Transwell assay (10^6^ cells/mL, 8-μm pore membranes, 3422, Corning, USA). For invasion, 5 mg/L matrigel (354230, BD Biosicences, USA) was precoated the membrane for simulating the extracellular matrix. Serum free medium was added in upper chamber while 10% FBS medium was added in lower chamber. After 24 h, the migrated or invaded cells on the bottom surface of membranes were stained with crystal violet, photographed by a microscope, and counted by using ImageJ 1.50b.

### Statistical analysis

All experiments were repeated three times and data were presented as mean ± standard deviation. Data obtained from different treatment groups were statistically compared with one-way ANOVA followed by Tukey’s test. Differences were considered significant at *P* < 0.05.
